# Pseudo-Sciatica Pain Due to a Biceps Femoris Muscle Tear

**DOI:** 10.7759/cureus.74286

**Published:** 2024-11-23

**Authors:** Rodrigo Correia, Guilherme Correia, Sofia Meixedo, Miguel de Castro Correia, José Luís Carvalho

**Affiliations:** 1 Physical Medicine and Rehabilitation, Centro de Reabilitação do Norte, Unidade Local de Saúde (ULS) Gaia/Espinho, Vila Nova de Gaia, PRT; 2 Orthopedics and Traumatology, Unidade Local de Saúde (ULS) Braga, Braga, PRT; 3 Intervention and Musculoskeletal Rehabilitation, Centro de Reabilitação do Norte, Unidade Local de Saúde (ULS) Gaia/Espinho, Vila Nova de Gaia, PRT

**Keywords:** differential diagnostic process, muscle injury, physical medicine and rehabilitation, sciatica pain, ultrasound imaging

## Abstract

Hamstring injuries (HSIs) are common among athletes, particularly in high-speed sports, and are frequently associated with prolonged recovery periods and a high recurrence rate. This study presents a 36-year-old male with sciatica-like symptoms following an acute hamstring tear during an amateur football match. The patient experienced this pain two weeks after the injury, suggesting potential sciatic nerve involvement. Ultrasonographic imaging revealed a partial myotendinous rupture of the biceps femoris long head, adjacent to the sciatic nerve. The patient underwent a structured five-week rehabilitation program, resulting in significant improvement, including reduced pain, enhanced muscle strength, and improved flexibility. This case highlights the role of early intervention and targeted rehabilitation in preventing complications, such as hamstring syndrome (HSS), and emphasizes the importance of using ultrasonography for diagnosis. This study also addresses the limitations in diagnostic imaging and provides insights into the link between muscle injury and sciatic nerve irritation, contributing valuable knowledge to the limited literature on this subject.

## Introduction

Hamstring injuries (HSIs) are among the most prevalent injuries affecting professional athletes, particularly in sports such as soccer (football), American football, and Australian rules football. These injuries are more frequently observed during competitive matches [1-8]. In 2016, Ekstrand et al. reported a 4% annual increase in the incidence of hamstring injuries [9]. The injury rate during competitive games was 4.77 per 1,000 hours, nine times higher than the 0.51 per 1,000 hours recorded during training sessions [9]. Notably, this annual increase in injuries was more pronounced during training [9].

Hamstring injuries account for over 39% of all reported sports-related injuries and can lead to significant time loss, ranging from 17 to 90 days. The recurrence rate for HSIs is notably high, between 12% and 63%. This high recurrence may be attributed, in part, to insufficient rehabilitation, failure to address the underlying etiology of the injury, or premature return to play (RTP) without meeting established criteria [10]. Prior injury is consistently identified as the most significant risk factor for HSIs in the literature [10]. HS strains occur more frequently in men [10].

Regarding injury mechanisms, hamstring strains are most likely to occur during high-speed activities such as sprinting, hurdling, kicking, and other movements that involve coupling hip flexion with knee extension [10,11]. These strains are categorized into two types as follows: type 1 injuries typically occur during running or sprinting, with the long head of the biceps femoris (BFlh) being the most elongated and, therefore, the most commonly injured muscle. Type 2 injuries result from excessive hamstring lengthening, particularly when the hip is flexed, and the knee is extended, as seen in activities like gymnastics, dancing, sliding, or tackling. This type of injury usually involves the semimembranosus (SM) and its proximal free tendon [9-11].

In professional soccer, an analysis of 180 hamstring strain injuries (HSIs) reported across 23 European clubs revealed that 84% involved the biceps femoris (BF), 11% affected the semimembranosus (SM), and 5% involved the semitendinosus (ST). The average recovery time from HSI was 36.7±19 days, highlighting the significance of injury location in determining prognosis and return-to-play (RTP) timelines [12]. According to some studies, recovery duration varies based on injury type as follows: myofascial injuries may require up to three weeks, myotendinous junction injuries four to eight weeks, and intratendinous injuries two to four months of conservative treatment [10].

Regarding treatment, a recent meta-analysis on the conservative management of HSI found that incorporating eccentric exercises can reduce the time to return to sport (RTS) and does not significantly affect the risk of re-injury [10].

In the available literature, it is empirically known that hamstring injuries near the sciatic nerve can lead to nerve irritation, resulting in neurological symptoms. In some cases, a traumatic lesion of the sciatic nerve can occur due to mechanical traction associated with muscle injury, or patients may develop hamstring syndrome (HSS) [13-15]. HSS was first described by Puranen and Orava in 1988 as a recurrent hamstring tear, causing deep pain at the ischial tuberosity and radiating discomfort in the posterior thigh [14]. The symptoms of HSS include pain at the ischial tuberosity, difficulty sitting for more than 30 minutes due to discomfort, and an increase in pain during the late swing phase of walking [14].

In the case presented in this article, the authors aimed to demonstrate how a muscle tear can lead to radicular pain, mimicking the symptoms of sciatica.

## Case presentation

A 36-year-old male salesman was referred to our consultation due to sciatica and gait pain. Upon thorough inquiry, he recounted the onset of pain two months prior during an amateur football match. The agony followed a forceful shot, accompanied by an abrupt sensation of a forceful "snap" in the hamstring area of his right leg and was unable to continue the game. He experienced an onset of electric shocks and tingling sensations that radiated from the back of his thigh down to his foot after a few weeks.

Physical assessment revealed an antalgic gait, as well as muscle wasting in the thigh and leg. Painful resistance during knee flexion was evident, alongside negative results for a straight leg raise (as the pain only manifested beyond a 60-degree elevation). The slump test produced uncertain findings. Further ultrasonographic investigation uncovered an immature proximal myotendinous rupture at the biceps femoris caput longus, proximate to the conjoined tendon. This rupture, involving 25% of the cross-sectional area, aligned with an adaptation of the classification of British Association of Muscle Injury Classification Ib (BAMIC Ib), and notably “sitting” upon the sciatic nerve.

Subsequently, the patient underwent a comprehensive five-week rehabilitation program (15 sessions, three times per week). The program encompassed therapeutic modalities to manage inflammation, soft tissue mobilization, and progressive strength training and flexibilization. The rehabilitation intervention was divided into three phases. Phase 1 - the primary goals were pain management, proper tissue healing, and restoring full range of motion. The patient successfully completed this phase in one week and was assessed before progressing to phase 2. Phase 2 - focus shifted to promoting optimal tissue healing, strengthening the muscles, and improving aerobic capacity/exercise tolerance. The patient completed this phase in two weeks, underwent evaluation, and advanced to the final phase. Phase 3 - this phase focused on scar tissue maturation, further muscle strengthening, enhancing aerobic endurance, and, most importantly, improving flexibility using the Askling L-protocol [16]. The patient completed this phase in two weeks.

**Figure 1:**
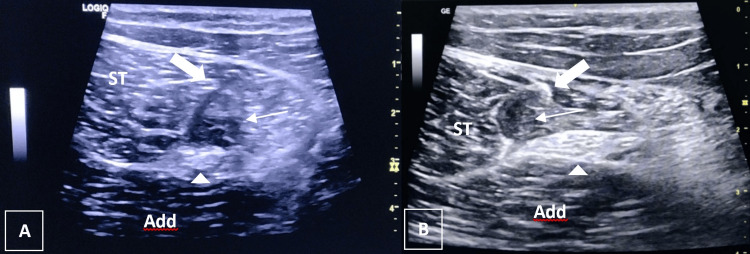
Ultrasound images of the presented case, before treatment (A) and after treatment (B). (A) Transverse scan of the proximal third of the posterior thigh with an immature myotendinous rupture with <25% of the cross-sectional area, adapted BAMIC Ib. (B) Transverse scan of the proximal third of the posterior thigh reveals advanced cicatrization. The thick arrow indicates the common tendon of the ST and biceps femoris long head, while the thin arrow points to the rupture, and the arrow-head represents the sciatic nerve. ST: semitendinosus; Add: adductor magnus; BAMIC Ib: British Association of Muscle Injury Classification Ib

In the conclusion of this intervention, the patient reported substantial improvement, characterized by the absence of sciatica pain and enhanced gait. Notably, pain only began during the terminal phase of a squat. The follow-up examination indicated reduced muscle wasting, negative outcomes in the resisted flexion test, and discomfort only upon muscle elongation. Ultrasonographic assessment corroborated these advancements, demonstrating more favorable signs of tissue healing and maturation (Figures 1A, 1B).

## Discussion

The patient presented with sciatica pain two months post-injury. Based on the mechanism of injury described, the most likely muscles affected are the semimembranosus (SM) or the long head of the biceps femoris (BFlh). Two weeks post-injury, the patient began experiencing radiculopathy-like symptoms, suggesting possible sciatic nerve involvement either at the time of injury or during the recovery process. The observed muscle atrophy during the examination can likely be attributed to disuse due to functional impairment, as the patient had been resting while awaiting consultation at our center.

During the physical examination, we performed Lasegue's test (straight leg raise) to assess for potential radiculopathy, alongside muscle strength evaluations, which showed preserved dorsiflexion and plantar flexion. Sensory function and osteotendinous reflexes were also intact, making radiculopathy less likely. To further investigate, we conducted the SLUMP test, which, despite eliciting only pain with stretching and mild tingling in the plantar region, suggested possible sciatic nerve involvement. Sonographic imaging revealed a BAMIC Ib lesion in the proximal long head of the biceps femoris (BFlh), positioned over the sciatic nerve, providing a more accurate explanation for the patient's symptoms. The inflammation around the injury site or a hematoma that may have developed two weeks post-injury could have caused compression of the sciatic nerve, leading to sciatica-like symptoms.

Given the injury had progressed over eight weeks without treatment, the patient was at high risk of developing hamstring syndrome (HSS) due to potential fibrosis around the sciatic nerve, which could result in nerve entrapment. To prevent this, we opted for a rehabilitation program at our center, allowing close monitoring and the possibility of addressing any entrapment with minimally invasive interventions.

The rehabilitation yielded positive results, with significant improvement in the patient's sciatica symptoms, muscle strength, and functional abilities, such as squatting. Additionally, hamstring flexibility improved. Emphasis was placed on elongation exercises, particularly using Askling's "L-protocol," which proved highly effective in promoting muscle flexibility and recovery [16].

The incidence of sciatic nerve involvement is not well-documented in the current literature. As noted by Puranen and Orava in 1988, even patients presenting with a positive Lasegue's sign showed no abnormal findings in electromyographic (EMG) examinations [14]. Therefore, clinical symptoms, physical examination, and imaging remain the primary diagnostic tools. A study by Martin et al. demonstrated that active knee flexion at 30º and 90º in the seated position yielded a sensitivity of 84% and specificity of 97% when evaluating 27 patients with proximal hamstring rupture [13].

Regarding treatment, the available literature is limited. Conservative management is generally preferred for partial ruptures, while surgical intervention is recommended for more severe cases, for instance, tendon avulsions and transverse tendon ruptures with retraction. However, the optimal timing for surgery remains unclear. Two systematic reviews provide conflicting results - Bodendorfer et al. and Harris et al. report better outcomes and higher patient satisfaction with acute repairs, whereas van der Made et al. found no significant difference between acute and delayed surgical repairs [15,17-19].

This study aimed to demonstrate that an acute muscle tear can be a potential cause of sciatic pain. In this instance, the condition was temporary due to timely and appropriate treatment. Without intervention, there could have been a risk of developing hamstring syndrome (HSS) later on. Given the scarcity of literature on this topic, the authors hope to contribute valuable insights.

The authors recognize some limitations in this report. Sonographic evaluations were conducted using different ultrasound systems due to equipment availability at the center. However, the provided images serve only illustrative purposes, as the primary value of ultrasound lies in dynamic evaluation and interpretation rather than a single static image. While a nerve conduction study or electromyographic assessment might have offered additional insights into sciatic nerve involvement, limited resources and high patient care demands prevented this. Additionally, the optimal timing for these tests may have been missed. Another limitation is the decision not to prescribe an MRI, which might have provided readers with a clearer view of the muscle injury and the extent of sciatic nerve involvement. However, ultrasound is sufficient for diagnosing muscle injuries and tracking nerves, and given the patient’s positive outcome following rehabilitation, the authors felt that further imaging would not add significant value. Therefore, they chose not to expend resources on tests that would likely confirm what was already evident.

## Conclusions

The combination of clinical evaluation and ultrasonographic imaging has emerged as a pivotal strategy in accurately diagnosing this complex condition. The positive outcome attained through tailored rehabilitation underscores the significance of personalized therapeutic strategies in optimizing patient recovery.
